# Fosfluconazole for Antifungal Prophylaxis in Very Low Birth Weight Infants

**DOI:** 10.1155/2009/274768

**Published:** 2009-04-23

**Authors:** Daijiro Takahashi, Tomohiko Nakamura, Reiko Shigematsu, Miyu Matsui, Shunsuke Araki, Kazuyasu Kubo, Hiroshi Sato, Akira Shirahata

**Affiliations:** ^1^Department of Pediatrics, School of Medicine, University of Occupational and Environmental Health, Kitakyushu 807-8555, Japan; ^2^Division of Neonatology, Nagano Children's Hospital, Azumino City, Nagano 399-8288, Japan

## Abstract

We conducted a retrospective case series study to evaluate the safety of fosfluconazole prophylaxis for preventing invasive fungal infection in VLBW infants with a central vascular access. Fosfluconazole was administered intravenously at a dose of 6 mg/kg everyday during which time a central venous catheter was placed. A total of 23 infants met the criteria for enrollment in our study. No cases of fungal infection were detected during the central venous catheter placement in the group. None of the infants had an elevated *β*-D-glucan, and all of them were still alive at discharge. Regarding the liver and renal function, no statistically significant differences were observed before and at the end of fosfluconazole prophylaxis. The results of this study demonstrate that fosfluconazole prophylaxis in preventing invasive fungal infection was well tolerated by VLBW infants. This is a first report to describe antifungal prophylaxis using fosfluconazole for VLBW infants.

## 1. Introduction

Very low birth weight (VLBW) infants are at risk for invasive fungal infection because of their immature immune system and the invasive supportive care they require [1]. Candida species rapidly colonize the skin and mucous membranes of about 60 percent of critically ill neonates during their first month in the neonatal intensive care unit (NICU), and they can progress to an invasive fungal infection in up to 20% of these infants [2]. Since the diagnosis and treatment are often delayed, recent research attention has focused on strategies to prevent such invasive fungal infection. Fluconazole is an antifungal agent which is efficacious for the treatment of serious systemic fungal infections. Prophylaxis with fluconazole reduces the incidence of colonization and invasive candida infection in neonates weighing less than 1500 g at birth [2, 3]. Kicklighter et al. reported that in infants with a birth weight <1500 g, fluconazole prophylaxis reduced the incidence of candidal colonization during the first month of life from 46% to 15%, a statistically significant reduction [4]. 

Fosfluconazole is a phosphate prodrug of fluconazole which is highly soluble (>100 mg/ml) compared with fluconazole (4 mg/ml). In vitro, fosfluconazole is at least 25-fold less potent than fluconazole against single isolates of *Candida* species and *Cryptococcus neoformans* but in vivo has similar efficacy to fluconazole [5]. These observations are consistent with efficient conversion of fosfluconazole to fluconazole in vivo.

Consequently, there is a need for a small volume formulation of fluconazole and the volume can be reduced using fosfluconazole. To date, there has been no report describing systemic antifungal prophylaxis using fosfluconazole for infants to assess the effect of this treatment modality on invasive fungal infection. The aim of our study was to evaluate the safety of fosfluconazole prophylaxis in preventing invasive fungal infection in VLBW infants with a central vascular access.

## 2. Material and Methods

We conducted a retrospective case series study to evaluate the safety of fosfluconazole prophylaxis in preterm infants. The study subjects consisted of VLBW infants with a central vascular access admitted to the NICU at the University of Occupational and Environmental Health between September 2006 to August 2007 and who were administered fosfluconazole for prophylaxis against invasive fungal infection. The data on the infants' demographic, clinical characteristics, and outcome were retrospectively collected during hospitalization and through a careful review of their medical records. 

We also evaluated the difference in the liver function (levels of serum aspartate aminotransferase (AST), and alanine aminotransferase (ALT)) and renal function (blood urea nitrogen and creatinine) before and at the end of the administration of fosfluconazole. In addition, we monitored the *β*-D-glucan levels when the central venous access was removed.

Fosfluconazole (Prodif) was administered intravenously at a dose of 6 mg/kg everyday during which time a central venous catheter was placed. *An invasive candida infection* was defined as the isolation of Candida from the peripheral blood, cerebrospinal fluid, or other normally sterile body fluid with clinical signs suggesting infection. A statistical analysis was performed using the *t*-test for nonparametric variables. A *P* value < .05 was considered to indicate statistical significance. *Before enrollment to this study, informed consent was obtained from each infant's parents or guardian*. This study was carried out under the control of the Ethics Committee of Medicine and Medical Care, University of Occupational and Environmental Health.

## 3. Results

A total of 23 infants met the criteria for enrollment in our study. None of the newborn infants was suspected of having a fungal infection at birth. The patients' demographic and neonatal characteristics and major risk factors for fungal infections are listed in Table 1. The mean gestational age was 28.8 weeks and birth weight was 1088 g.

No cases of fungal infection were detected during the central venous catheter placement in the group. None of the infants had an elevated *β*-D-glucan, and all of them were still alive at discharge. Table 2 shows the comparison of liver aminotransferase levels and the renal function before and at the end of fosfluconazole prophylaxis. No statistically significant differences were observed, but the ALT tended to be higher at the end of the treatment. At the time when the central venous catheter was removed, elevations in the serum AST and ALT levels of more than twice the normal range were recorded in 1 neonate. No serious adverse events or fosfluconazole-related toxic effects were recorded. Drug administration was not discontinued because of any presumed adverse events, intolerance or potentially dangerous interactions with other drugs.

## 4. Discussion

*This study was a case series of VLBW infants with a central venous access treated with fosfluconazole who did not demonstrate any obvious adverse effects on either the transaminase levels or the renal function at the dosage used.*

A low-volume product would allow for the bolus administration, reducing fluid and sodium load, and it could also facilitate access to higher doses. The current intravenous dosage form requires, for example, with fluconazole a high-volume infusion which is undesirable in critically ill patients. A smaller, concentrated product would therefore offer advantages to the patient in clinical use, especially for VLBW infants.

An evaluation of the adverse effects among VLBW infants is extremely difficult because these patients are usually both primarily very sick while they also often receive several different drug therapies concomitantly. In addition, many of the adverse effects of fosfluconazole could not be evaluated in newborns. 

Our study demonstrated that an asymptomatic mild elevation of ALT occurred. Mild and transient increases of liver enzymes, without clinical implications, have previously been described in infants receiving fluconazole [3, 4], and therefore a transient increase in the hepatic aminotransferase levels may occur similarly when using fosfluconazole in VLBW infants. Regarding the renal function, no obvious renal toxicity was observed in our study.

The use of antifungal prophylaxis is associated with the potential risk of selecting resistant organisms. This may take the form of either a subtle increase in the minimal inhibitory concentrations of previously sensitive species or an increased incidence of inherently resistant species. We did not investigate drug resistance in this study; therefore, we would like to study this issue in a future study.

Furthermore, this study was a retrospective and observational study that consisted of only a small number of participants; therefore, a prospective controlled randomized clinical trial on the use of fosfluconazole for prophylaxis in VLBW infants is called for in the future.

## Figures and Tables

**Table 1:** Characteristics of the patients' risk factors for invasive fungal infection.

Baseline characteristics (*N* = 23)		
* *Birth weight-g		
* * * *Mean (range)	1088 ± 299	(615–1466)
* *Gastational age-wk		
* * * *Mean (range)	28.8 ± 3.3	(24.5–36.5)
* *Male/Female sex-no.	12/11	
* *Antinatal steroids-no. (%)	13	(57)
* *Antinatal antibiotics-no. (%)	9	(39)
* *Rupture of membranes ≧24 hr		
* * * *before delivery-no. (%)	4	(17)
* *Born at study hospital-no. (%)	22	(96)
* *Vaginal delivery-no. (%)	5	(22)
* *APGAR score		
* * * *1 min	5.4 ± 2.0	
* * * *5 min	7.6 ± 2.0	
* *Age at enrollment		
* * * * <24 h	18	
* * * *24–48 h	5	
* *Duration of prophylaxis		
* * * *Mean (range)	10.3 ± 7.8	(3–35)
* *Treatment received-no. (%)		
* * * *Steroids	6	
* * * *Ampicillin and Aminoglycosides	19	
* * * *Cephalosporins	2	
* * * *Carbapenems	2	
* *Duration of antibiotics therapy-days		
* * * *Mean (range)	5.8 ± 1.9	(4–12)
* *Vascular access-no. (%)		
* * * *Central vein	23	(100)
* * * *Umbiliical	1	(4)
* * * *Peripheral arterial	8	(35)

* ± values are means ± SD. **The APGAR score ranges from 0 to 10, with higher scores indicating better functioning.

**Table 2:** Comparison of the liver aminotransferase and renal function before and at the end of fosfluconazole prophylaxis.

	At enrollment	At the end of the treatment	*P*-value
AST	45.5 ± 32.2	35.9 ± 10.6	n.s.
ALT	4.3 ± 2.3	10.8 ± 17.5	n.s.
BUN	10.2 ± 5	9.5 ± 5.3	n.s.
Creatinine	0.7 ± 0.6	0.8 ± 0.1	n.s.

AST, aspartate aminotransferase; ALT, alanine aminotransferase; BUN, blood urea nitrogen.

## Abbreviations

VLBW: Very-low-birth-weight
NICU: Neonatal intensive care unit
AST: Aspartate aminotransferase
ALT: Alanine aminotransferase
